# A Simulation-Based Study on the Comparison of Statistical and Time Series Forecasting Methods for Early Detection of Infectious Disease Outbreaks

**DOI:** 10.3390/ijerph15050966

**Published:** 2018-05-11

**Authors:** Eunjoo Yang, Hyun Woo Park, Yeon Hwa Choi, Jusim Kim, Lkhagvadorj Munkhdalai, Ibrahim Musa, Keun Ho Ryu

**Affiliations:** 1 Emergency Operations Center, Centers for Disease Control and Prevention, Cheongju 28644, Korea; ej.yang@korea.kr (E.Y.); cyh6803@korea.kr (Y.H.C.); jskim01@korea.kr (J.K.); 2 College of Electrical and Computer Engineering, Chungbuk National University, Cheongju 28644, Korea; hwpark@dblab.chungbuk.ac.kr (H.W.P.); lhagii@dblab.chungbuk.ac.kr (L.M.); ibrahim@dblab.chungbuk.ac.kr (I.M.)

**Keywords:** syndromic surveillance, outbreak detection, aberration detection, syndromic diarrhea

## Abstract

Early detection of infectious disease outbreaks is one of the important and significant issues in syndromic surveillance systems. It helps to provide a rapid epidemiological response and reduce morbidity and mortality. In order to upgrade the current system at the Korea Centers for Disease Control and Prevention (KCDC), a comparative study of state-of-the-art techniques is required. We compared four different temporal outbreak detection algorithms: the CUmulative SUM (CUSUM), the Early Aberration Reporting System (EARS), the autoregressive integrated moving average (ARIMA), and the Holt-Winters algorithm. The comparison was performed based on not only 42 different time series generated taking into account trends, seasonality, and randomly occurring outbreaks, but also real-world daily and weekly data related to diarrhea infection. The algorithms were evaluated using different metrics. These were namely, sensitivity, specificity, positive predictive value (PPV), negative predictive value (NPV), F1 score, symmetric mean absolute percent error (sMAPE), root-mean-square error (RMSE), and mean absolute deviation (MAD). Although the comparison results showed better performance for the EARS C3 method with respect to the other algorithms, despite the characteristics of the underlying time series data, Holt–Winters showed better performance when the baseline frequency and the dispersion parameter values were both less than 1.5 and 2, respectively.

## 1. Introduction

A number of emergency department-based syndromic surveillance systems and early warning systems for early detection of adverse disease events have been implemented since the year 2000. Syndromic surveillance is defined as the ongoing systematic collection, analysis, and interpretation of “syndrome”-specific data for early detection of public health aberrations [1]. The Korea Centers for Disease Control and Prevention (KCDC) has also implemented an emergency department-based syndromic surveillance system. The system was designed to identify illness clusters before diagnoses are confirmed and reported to public health agencies. The system is connected to a considerable number of emergency departments from 17 provinces and cities in Korea and has been used to monitor the daily status of five different syndromes.

The incidence of infectious disease has been growing in relation to population growth, density, and climate change [2,3]. Therefore, a number of statistical methodologies have been proposed for syndromic surveillance systems based on the collected historical time series data using a computer database system. These statistical methodologies can be classified into two major areas such as statistical detection and time series forecasting methods [4,5].

To design upgraded and enhanced functions of the system for the early warning of adverse disease events, we survey many suitable algorithms for syndromic surveillance, and analyze and evaluate some algorithms. According to our survey, the CUmulative SUM (CUSUM) [6,7] and the Early Aberration Reporting System (EARS) [7] statistical detection approaches and the autoregressive integrated moving average (ARIMA) [8] and Holt-Winters [9,10] time series forecasting approaches have been used in a large number of papers and applications.

Ronald et al. [7] applied the CUSUM algorithm to adaptive regression residuals and compared it with EARS (C1, C2, and C3) based on a simulation data for syndromic surveillance. In their study, the CUSUM method with adaptive regression residuals showed better results than EARS. Recently, Gabriel et al. [11] compared 21 statistical algorithms for temporal outbreak detection. However, these studies did not include time series forecasting methods.

Allard et al. [5] and Reis et al. [12] presented the integer-valued autoregressive (INAR) and ARIMA time series models for a syndromic surveillance system. Howard et al. [13] compared three different time series models, namely the non-adaptive regression, adaptive regression, and Holt-Winters approaches utilizing real bio-surveillance time series.

Unfortunately, those studies did not compare both the statistical detection and time series forecasting algorithm categories. Therefore, the aim of this study is to define the advantages and disadvantages of these methods based on time series data on trends, seasonality, and random outbreaks.

In order to address the aforementioned issues, this paper carries out an experimental study based on simulated time series to evaluate state-of-the-art algorithms. In addition, it contributes a limited case of real-world data related to diarrhea infection. Towards achieving these goals, simulated time series data based on a negative binomial method which takes into account not only trends and seasonality, but also randomly occurring outbreaks were generated guided by the recent work of Angela et al. [14]. On the other hand, because the methodologies considered in this study generally fall into two main categories (statistical and forecasting methods), different evaluation metrics were used.

Statistical detection methods are commonly evaluated using: true positive rate (sensitivity), true negative rate (specificity), positive predictive value (PPV), negative predictive value (NPV), and F1 score. In contrast, time series forecasting methods were mostly evaluated by the root-mean-square error (RMSE), mean absolute percentage error (MAPE) and mean absolute deviation (MAD). Therefore, this study used: PPV, NPV, F1 score, a “symmetric” mean absolute percentage error (sMAPE), RMSE, and MAD.

This paper is organized as follows. We briefly present a description of the evaluation framework, selected methods, and evaluation metrics used to compare the algorithms in Section 2. Section 3 presents the process of simulating the data and evaluating the experimental results. Finally, Section 4 concludes the experimental result and comparison, as well as discussing the advantages and disadvantages of the selected methods.

## 2. Methods and Materials

### 2.1. Outbreak Detection Algorithms and Evaluation Metrics

**Figure 1:**
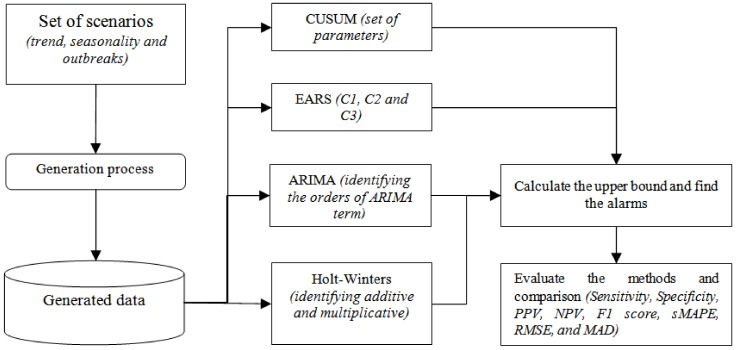
Framework for comparing the outbreak detection algorithms. EARS: Early Aberration Reporting System; ARIMA: auto regressive integrated moving average; PPV: positive predictive value; NPV: negative predictive value; sMAPE: symmetric mean absolute percent error; RMSE: root-mean-square error; MAD: mean absolute deviation; CUSUM: CUmulative SUM.

The overall framework of this study is shown in Figure 1. Firstly, we generated time series data using several combinations of seasonality and trends for syndromic surveillance following the same procedure as in Angela et al. [14]. In the second part, four different approaches of syndromic surveillance (CUSUM, EARS, ARIMA, and Holt-Winters) were analyzed using the generated data. Finally, the four approaches were compared using the selected evaluation metrics. Also, real-life diarrhea syndromic surveillance data were used in the final step where daily and weekly data were fed into the selected algorithms in order to detect alarms related to diarrhea infection. However, since the real-world data of the syndromic diarrhea did not include an attribute that defined the state of the outbreak, the performances of the algorithms in this case were evaluated based on sMAPE, RMSE, and MAD as evaluation metrics.

In order to implement the widely used CUSUM algorithm developed in 1954 by Page et al. [15], as well as variants of the EARS algorithm, the “R surveillance package” [16]—a popularly used open source toolkit—was used. The package comprised assorted algorithms such as standardized variables transformations (anscombe and rossi) [6], and the generalized linear model (glm) [17] for time-varying expectations. The analysis was carried out based on the default settings incorporated in the package.

In the case of the ARIMA [18] and Holt-Winters methods [19], a different package was used, namely, the R forecast package which is dedicated to time series forecast analysis [20,21]. The package is sophisticated enough to automatically select between ARIMA and Holt-Winters as forecasting models depending on whether the time-series includes trends or seasonality.

As referred in the introduction, the previous related studies mostly used sensitivity, specificity, PPV, NPV, and F1 score as evaluation metrics to measure the performances of outbreak detection algorithms, and RMSE, MAPE, and MAD metrics for time series forecasting methods. Particularly, RMSE, MAPE and MAD metrics were found to be suitable when the datasets did not include a variable for the state of the outbreak. However, due to the fact that even with the existence of outbreak state in the data the results of sensitivity, specificity, PPV, NPV, and F1 are inconsistent and disagreeable, in this paper, we applied the “symmetric” MAPE (sMAPE) method proposed by Armstrong which was used in an M3 forecasting competition [22]. Therefore, the evaluation metrics used in this paper are: sensitivity, specificity, PPV, NPV, F1 score, sMAPE, RMSE and MAD.

### 2.2. Data Simulation

This section describes the details of generating the simulated data. In essence, the data generation process was carried out in a similar way to that of Angela et al. [14]. In the process, baseline time series counts without outbreaks were generated based on a negative binomial model of mean (μ) and variance (ϕμ) with a dispersion parameter (ϕ).

The formula for the baseline simulation is defined as:

$$\log\left(\mu_{t}\right)=\theta +\beta t+\overset{m}{\underset{j=1}{\sum }}\left(\gamma_{1}\cos\left(\frac{2\pi jt}{52}\right)+\gamma_{2}\sin\left(\frac{2\pi jt}{52}\right)\right)$$

where theta (θ) is the baseline frequency of reports, beta (β) is the time trend, gamma1 ($\gamma_{1}$) is the seasonality, gamma2 ($\gamma_{2}$) is the biannual seasonality, m = 0 formally corresponds to no seasonality, m = 1 corresponds to annual seasonality, and m = 2 shows biannual seasonality.

The time series generation process was guided by 42 different parameter combinations as shown in Table S1 in the Supplementary Materials. For each case of these parameter combinations the simulation was replicated 100 times to generate data for *n* = 624 weeks in each individual run. Thereafter, in each replica, the simulated data were split into three parts. The first part spans the period from week 1 to week 312 to represent training time series, followed by the data from week 313 through week 575 as the baseline, and the final data from week 576 to week 624 were left to resemble time series of the current week.

Outbreaks in baseline weeks: For each scenario, we included four outbreaks with start times chosen randomly among the baseline weeks (weeks 313–575). We took the values of k to be 2, 3, 5 or 10. Where the parameter k resembles the number of outbreaks.

Outbreaks in current weeks: For each scenario, we included one outbreak with start time chosen randomly among the last 49 weeks (weeks 576–624). We choose the values of k to be in the range 1–10.

**Figure 2:**
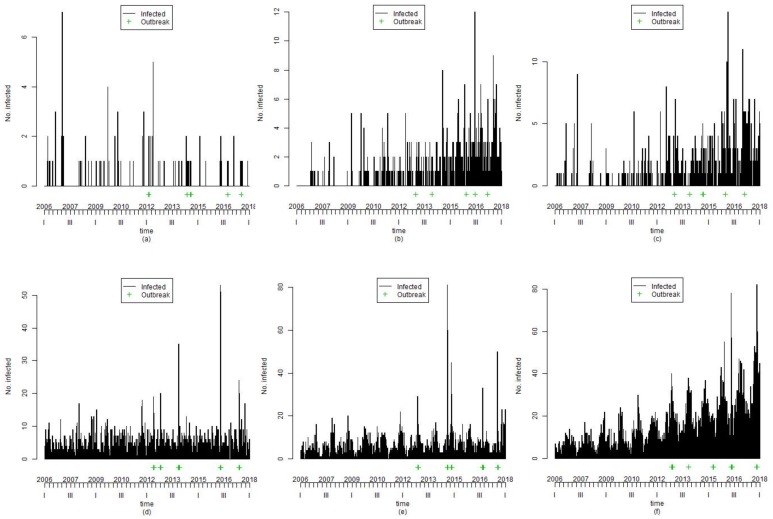
Examples of simulated data: (**a**) Scenario 8; (**b**) Scenario 10; (**c**) Scenario 12; (**d**) Scenario 13; (**e**) Scenario 15; and (**f**) Scenario 17. In each graph, the x-axis represents time, the y-axis represents the number of infections, and the green-colored plus sign resembles outbreaks.

Finally, because of this data generation, 4200 series were prepared to analyze the outbreak detection for the syndromic surveillance system. The value 4200 was obtained by multiplying the 42 scenarios by 100 replications. As an example of the data generated by the above-mentioned procedure, we present the data of six selected scenarios as follows: (1) Scenario 8 comprises the baseline frequency (θ = −2), and annual seasonality (m = 1 and $\gamma_{1}=0.1$); (2) Scenario 10 comprises the baseline frequency (θ = −2), and trend (β=0.005); (3) Scenario 12 comprises the baseline frequency (θ = −2), trend (β=0.005), and biannual seasonality (m = 2, $\gamma_{1}=0.1$ and $\gamma_{2}=0.3$); (4) Scenario 13 comprises only the baseline frequency (θ = 1.5); (5) Scenario 15 comprises the baseline frequency (θ = 1.5), and biannual seasonality (m = 2, $\gamma_{1}=0.2$ and $\gamma_{2}=-0.4$); and (6) Scenario 17 comprises the baseline frequency (θ = 1.5), trend (β=0.003), and annual seasonality (m = 1 and $\gamma_{1}=0.2$), as shown in Figure 2. In the figure, each graph of a scenario consists of an x-axis, which represents time, a y-axis, which represents the number of infections, a line that represents infections, and a green-colored plus sign to represent outbreaks.

## 3. Results

### 3.1. The Results of CUSUM

The underlying mechanism of the CUSUM algorithm is to account for the accumulation of deviations between previous and current values of a given time series. Since the algorithm needs a guide to count the deviations, it requires a parameter, h, to show the upper bound of the time series, and an additional parameter, k, to represent the tolerated shift from the mean of the underlying monitored time-series. For the purposes of our experiments, the default settings of these parameters in the “R surveillance package” were used. However, since the default parameters assume a time series without trends and seasonality, in this paper, trend and seasonality were added into a generalized linear model resulting in a new model named “glm with trend” which requires an additional parameter, namely, “trans = ‘rossi’”, which indicates a version of the CUSUM algorithm that deals with trends, and seasonality [6]. Then, we calculated the evaluation metrics for each of the 42 scenarios in a similar way to that of Angela et al. [14].

**Figure 3:**
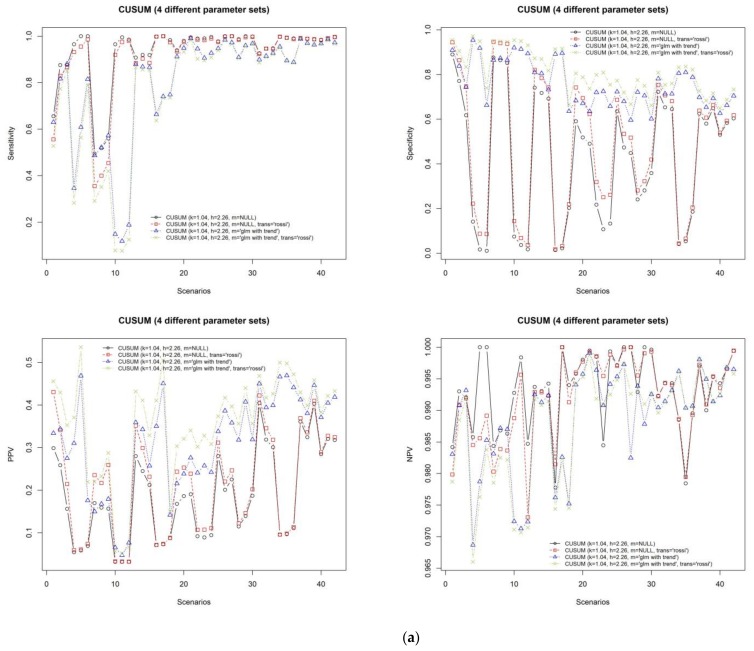

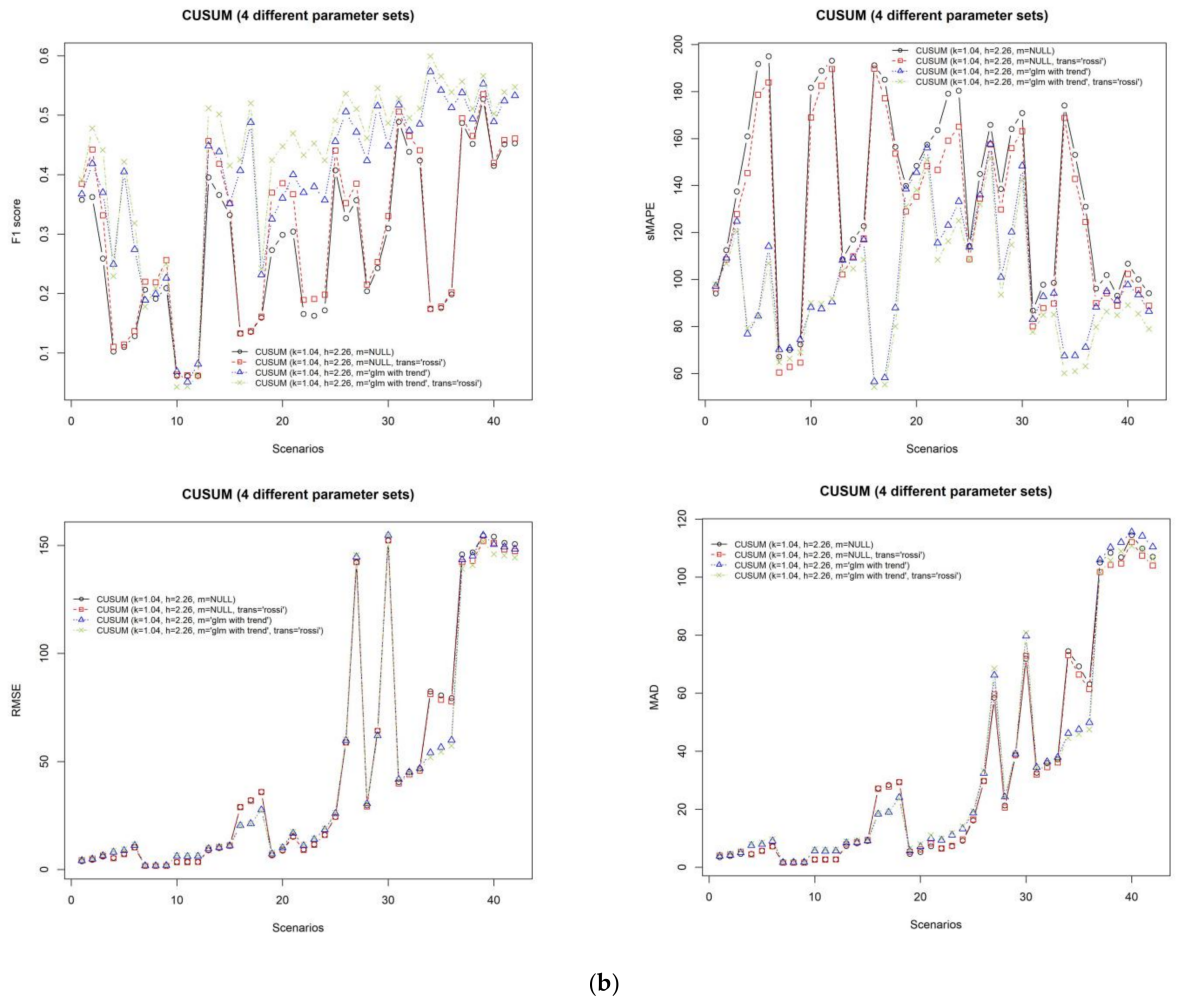
(**a**) The results of the four different CUSUM algorithms with different sets of parameters in terms of sensitivity, specificity, PPV, and NPV across the all scenarios. (**b**) The results of the four different CUSUM algorithms with different sets of parameters in terms of F1 score, sMAPE, RMSE, and MAD across the all scenarios. CUSUM: the CUmulative SUM.

In order to determine which parameter configuration will result in a better performance of CUSUM, the performances of the four sets of parameters are measured by the eight different evaluation metrics as illustrated in Figure 3a,b. The “rossi” and “glm with trend” (k = 1.04, h = 2.26, m = “glm with trend” and trans = “rossi”) algorithms showed good results compared with other CUSUM algorithms.

For the best CUSUM algorithm, average sensitivity is equal to 0.77, specificity is equal to 0.81, PPV is equal to 0.36, and NPV is equal to 0.99. According to this result, although it provides many false alarms, the true outbreak detection rate is high. Also, its average F1 score is equal to 0.424. The F1 score shows the trade-off between sensitivity and PPV. If the F1 score is close to 1, it shows good performance in detecting the outbreaks without false alarms.

The metrics of RMSE and MAD are meant to measure the differences between the predicted number of infections by a given outbreak detection algorithm and the actual number of observed infections. Higher values of these metrics indicate poor performance of a given algorithm. From Figure 3a,b, however, it is evident that even though the values of these two metrics were slightly higher, the algorithm still revealed good performances in terms of sensitivity, specificity, PPV, NPV, and F1 score. Therefore, we suggested sMAPE evaluation metric to address this problem. When the sMAPE value is closer to 0, it indicates that the selected method shows good performance. Additionally, this evaluation metric is agreeable to evaluate the selected methods because the correlation between F1 score and sMAPE was −0.7. This means that if the F1 score is high, sMAPE will be low.

The average sMAPE of the best CUSUM algorithms is equal to 95.76, the average RMSE is equal to 45.16, and MAD is equal to 32.58.

### 3.2. The Results of EARS C1, C2 and C3

The C1, C2, and C3 of the EARS are among the most commonly used syndromic surveillance techniques. Although they were meant to follow similar detection approach like CUSUM, they instead compute number of counts from the recent past [7]. In addition, C1, and C2 utilize Shewhart control charts that use a moving sample average and sample standard deviation to standardize each observation.

**Figure 4:**
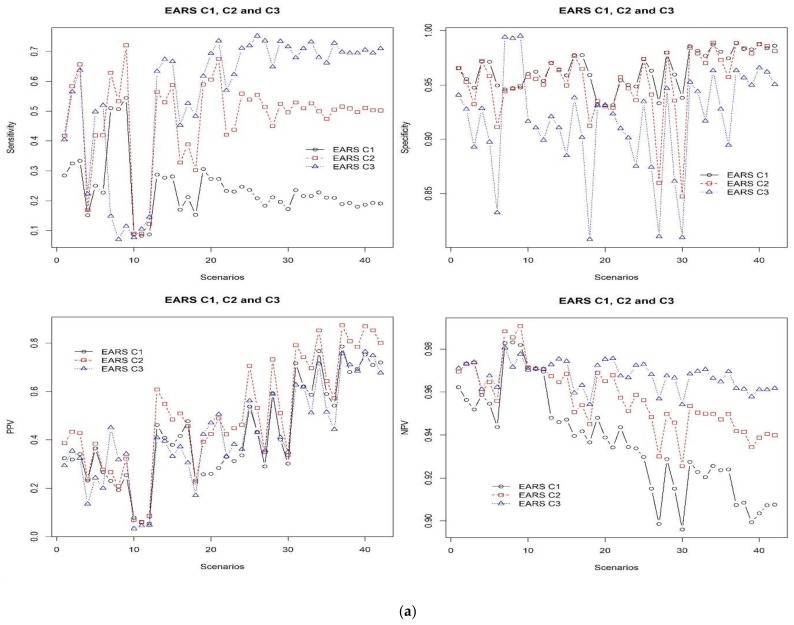

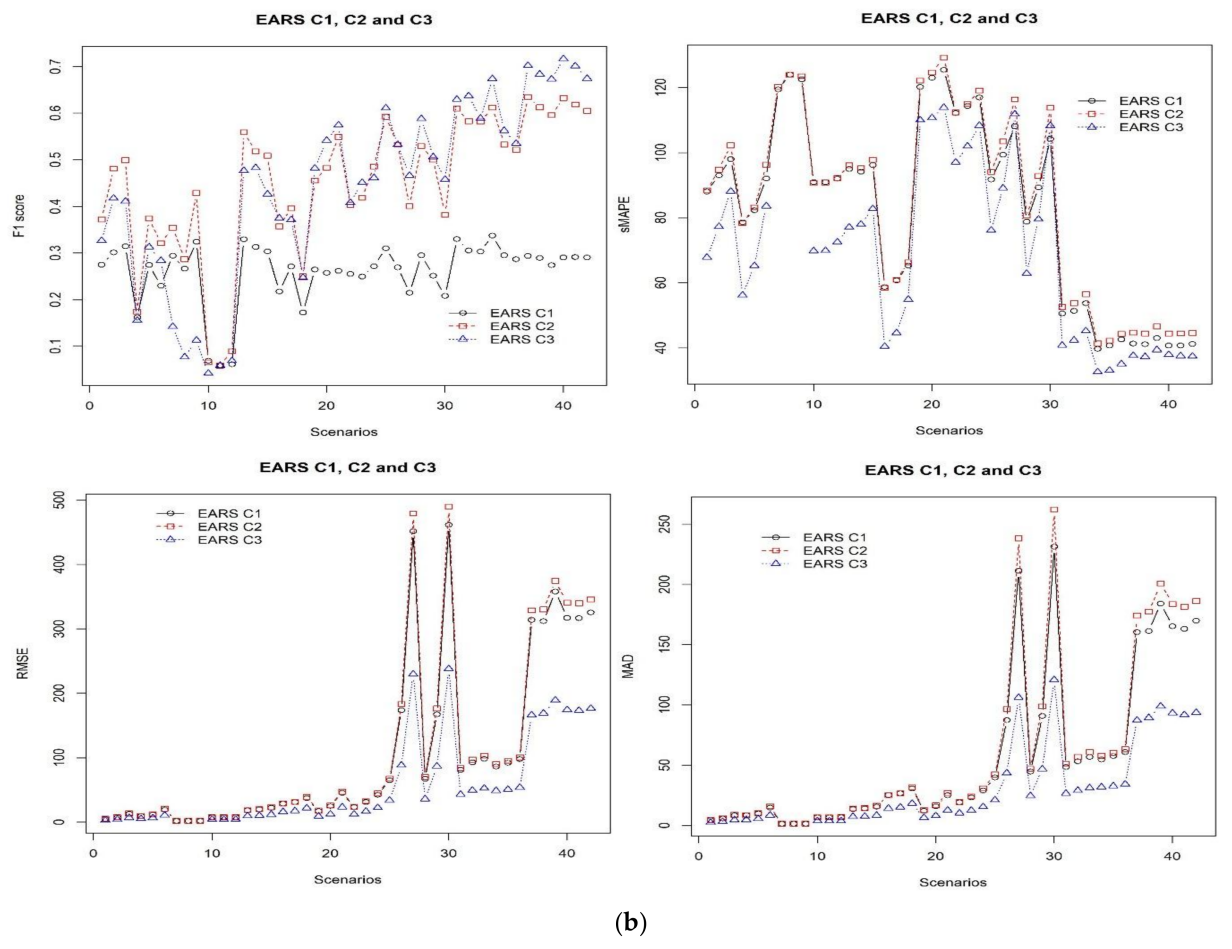
(**a**) The results of comparing EARS C1, C2, and C3 in terms of sensitivity, specificity, PPV, and NPV across the all scenarios. EARS C3 showed better performance in almost all cases. (**b**) The results of comparing EARS C1, C2, and C3 in terms of F1 score, sMAPE, RMSE, and MAD across the all scenarios. EARS C3 showed better performance in almost all cases as it showed lower values of sMAPE, RMSE, and MAD, and higher values of F1 score.

In order to evaluate these methods, the R surveillance package was used and the performances were observed and reflected in Figure 4a,b. Figure 4a shows the results of sensitivity, specificity, PPV, and NPV when C1, C2, and C3 were compared using the synthetic dataset across all of the scenarios. Equally, in terms of the performance values of F1 score, RMSE, SMAPE, and MAD, EARS C3 was the best method, as shown in Figure 4b.

### 3.3. Performance Evaluation of CUSUM, EARS C3, ARIMA and Holt-Winters

**Figure 5:**
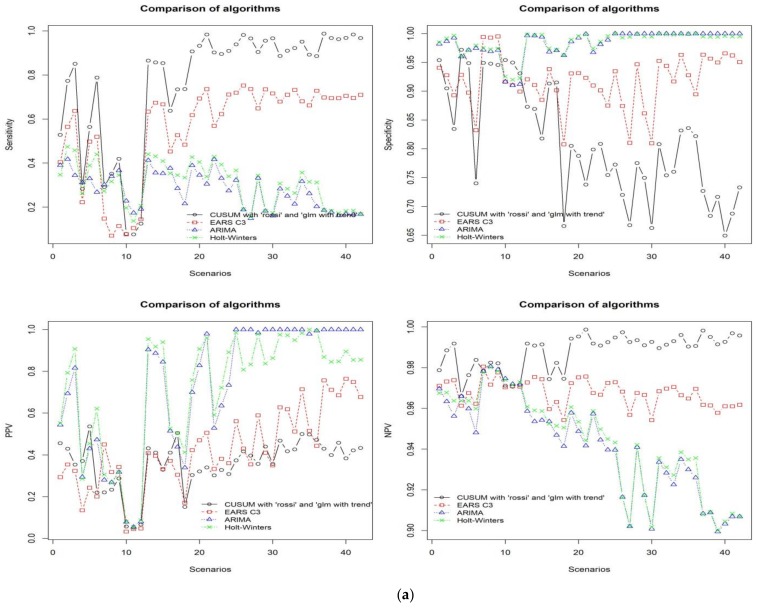

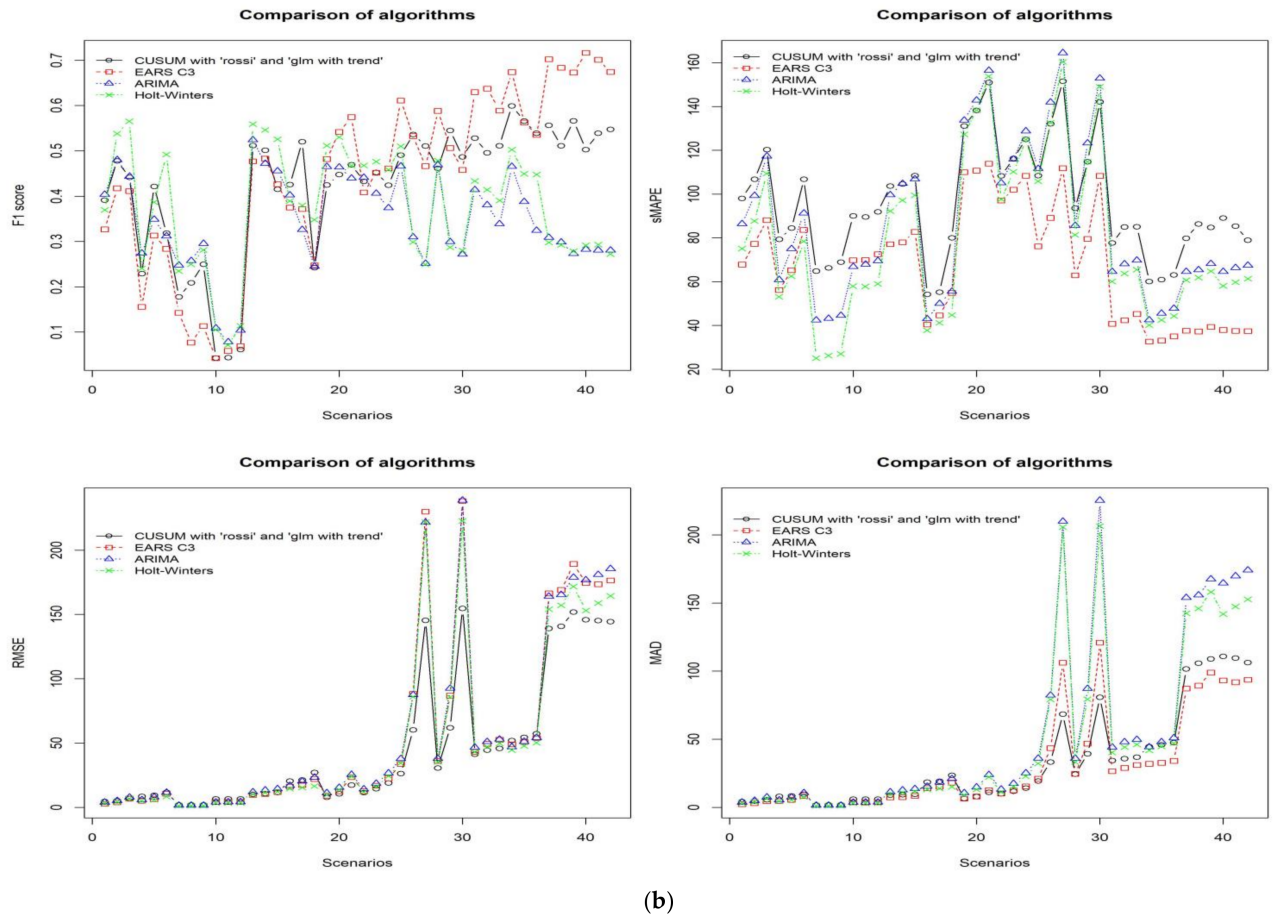
(**a**) The results of comparing CUSUM, EARS C3, ARIMA, and Holt-Winters in terms of sensitivity, specificity, PPV, and NPV across the all scenarios. (**b**) The results of comparing CUSUM, EARS C3, ARIMA, and Holt-Winters in terms of F1 score, sMAPE, RMSE, and MAD across the all scenarios.

In Section 3.1 and Section 3.2 the best settings required for CUSUM algorithm and EARS C3 to perform well are demonstrated. In this section, the all algorithms considered in this paper are compared. Namely, we compared the CUSUM, EARS C3, ARIMA, and Holt-Winters approaches. ARIMA and Holt-Winters are widely used for the purposes of time series forecasting in different research domains. The ARIMA algorithm requires parameters such as the autoregressive (AR), moving average (MA), and knowledge of whether the mode of its operation is integrated or not. On the other hand, Holt-Winters algorithm only requires the definition of additive and multiplicative seasonality. Fortunately, however, the underlying forecast library of the R language includes a function that automatically selects the ARIMA or Holt-Winter algorithm based on the underlying characteristics of the time series [21]. Figure 5a,b show the performance results of the four algorithms using the simulated data set across all scenarios. The graphs in Figure 5a show the comparative results of the algorithms in terms of sensitivity, specificity, PPV, and NPV. This figure shows that although the best setting for CUSUM shows outstanding results in terms of sensitivity and NPV, specificity and PPV were comparatively low. In contrast, both ARIMA and Holt-Winters achieved higher average values in terms of specificity (0.984, and 0.985) and PPV (0.941 and 0.944), respectively, and lower average values in terms of sensitivity (0.277 and 0.305), and NPV (0.729 and 0.718), in that order. Figure 5b shows the comparison of the algorithms in terms of F1 score, sMAPE, RMSE, and MAD. From these graphs the ARIMA and Holt-Winters approaches did not show competing performance against EARS C3 and CUSUM.

EARS C3 indicates average results for sensitivity, specificity, NPV, and PPV. Even though EARS C3 showed fewer false alarms than the best CUSUM, its true outbreak detection rate is low. However, the EARS C3 algorithm is the best approach to detect outbreaks for average F1 score and sMAPE metrics.

We also compared the methods depending on the characteristics of time series data as shown in Table 1. Our generated data can be divided into six groups based on the combination of scenarios. In this case, EARS C3 model also showed the best performance for all groups, except the time series with the trend and annual seasonality grouped according to F1 score and sMAPE metrics. The best CUSUM (rossi and glm with trend) algorithm indicated the best performance for the remaining group.

In Table 2, the data is divided into seven groups based on baseline frequency and dispersion parameters. ARIMA and Holt-Winter showed good performance when the baseline frequency was less than 1.5 and the dispersion was parameter less than 2. EARS C3 performed well in other cases.

### 3.4. Comparison of Selected Methods for Diarrhea Syndromic Surveillance

In this section, we run the selected algorithms using real world syndromic diarrhea surveillance data (2013–2017) obtained from the Korea Centers for Disease Control and Prevention. Datasets of the years 2013 through 2016 were used as a baseline data. The data from 2017 were designated as current data in order to evaluate the algorithms. Daily and weekly data are illustrated in Figures S1 and S2 in the Supplementary Materials. Figures S3 and S4 in the Supplementary Materials show the experimental results. In each figure the dashed blue line indicates an upper bound calculated by the underling algorithm. This is there to indicate if the current value referred to in the figure by the black lines surpassed this upper bound, and an alarm was raised. The small red triangular shapes indicate the alarm. In Figures S3 and S4, the black lines represent the actual cases of infectious diarrhea. The dashed-blue line indicates the upper bound calculated by the algorithm. Therefore, whenever number of actual infectious diarrhea surpasses the upper-bound, an alarm is raised, indicating a possibility of an outbreak. In this case, however, since the real-life diarrhea syndromic surveillance data do not include outbreak variable, the sMAPE, RMSE, and MAD evaluation metrics were used in this section. Fortunately, we found that the sMAPE metric is highly correlated with F1 score from the analysis of simulated data. According to sMAPE metric, the result of real data analysis showed same performance as the simulated data. EARS C3 also showed the best performance, as shown in Table 3. The result of the CUSUM (rossi and glm with trend) algorithm evaluated by RMSE and MAD indicated outstanding performance.

## 4. Discussion

The aim of surveillance system is to provide two main objectives, which are to maximize true outbreak detection rate and minimize false alarm rate. We analyzed most widely used four algorithms for early detection of infectious disease outbreaks in public health using simulated and real data. In order to compare the performances of the selected algorithms, we used several evaluation metrics. However, although algorithms that provide high sensitivity such as CUSUM and EARS were able to detect the majority of the outbreaks, algorithms that showed high specificity or PPV such as ARIMA and Holt-Winters detected fewer outbreaks. Furthermore, none of the algorithms considered in this study showed good results consistently across the all evaluation metrics.

Nevertheless, the F1 score metric demonstrates the trade-off between sensitivity and specificity in the surveillance system. Also, we compared sMAPE, RMSE and MAD evaluation metrics for time series forecasting methods to the F1 score, and the sMAPE is highly correlated with the F1 score. The EARS C3 algorithm’s performance, which was evaluated by the F1 score and sMAPE, was better than other algorithms for most of the scenarios.

In the CUSUM approach, we chose four different algorithms and set the default h and k parameters in a similar way as in [11]. However, the one difference is that we added the trend variable into “glm” model of CUSUM. Thereafter, the result of CUSUM (rossi and glm with trend) algorithm was much improved, such that average specificity increased by 0.33, PPV increased by 0.14, F1 score increased by 0.12, and sMAPE decreased by 32.6. The best CUSUM algorithm showed better results than the ARIMA and Holt-Winter algorithms and comparable results with the EARS C3 algorithm. In addition, the CUSUM model indicated the best performance when the data has trend and annual seasonality.

Practically, the CUSUM algorithm is good for detecting disease outbreaks, but it provides many false alarms in normal conditions except when the data has trends and annual seasonality. This is the disadvantage of CUSUM algorithm. The aim of an early outbreak detection method is to identify the largest possible number of outbreaks without false alarms [11].

For the ARIMA and Holt-Winters algorithms, the F1 score and sMAPE are lower than other algorithms. However, these algorithms can detect outbreaks very well on data that have a baseline frequency less than 1.5 (θ < 1.5) and a dispersion parameter of less than 2 (ϕ < 2). This means that these algorithms are more suitable to detect disease outbreaks that rarely cause infections in public health.

This study analyzed real data collected from the KCDC. The data represent historical cases of infectious diarrhea between 2013 and 2017. The results of the selected algorithms are similar to the simulated data because the real data show no trends and biannual seasonality.

## 5. Conclusions

This study compared the most useful four methods for early outbreak detection in syndromic surveillance systems based on multiple types of simulated data and real data. In the results, we used several evaluation metrics to compare the selected algorithms, but it is difficult to compare the selected algorithms because those algorithms have their own advantages and disadvantages. The four algorithms are compared according to the time series data type (trends, seasonality, and baseline infections), and the algorithms can be selected according to various data types.

The main conclusions from the results are that CUSUM (rossi and glm with trend) showed better performance compared to the other variants of CUSUM algorithm, particularly when the data show trends and annual seasonality. Additionally, if the baseline for infectious disease is less than 1.5 and its dispersion is small, ARIMA and Holt-Winter algorithms are good approaches to detect outbreaks of this kind of disease. For the EARS C1, C2, and C3 algorithms, C3 showed better performance than the other EARSs. It also showed good performance compared with other algorithms for the most of the scenarios. However, the EARS C3 algorithm is more suitable for outbreak detection when it considers both true outbreak detection rate and false alarm rate. Finally, we studied the selected approaches on the real data and the results were same as for the simulated data.

The contributions of this study are that we suggest sMAPE evaluation metrics for evaluating the performance of syndromic surveillance analysis when the data do not include the outbreak state variable, and we demonstrated that the “glm with trend variable” CUSUM algorithm is better than other default CUSUM algorithms.

## Figures and Tables

**Table 1:** The comparison of average performances for each type of time series.

Data Type and # of Scenarios	Method	Sensitivity	Specificity	PPV	NPV	F1 Score	sMAPE	RMSE	MAD
no trend and no seasonality (#1, #7, #13, #19, #25, #31, #37)	CUSUM	**0.77**	0.84	0.38	**0.99**	0.44	94.78	**33.04**	25.24
	EARS C3	0.56	0.95	0.50	0.97	**0.48**	**68.22**	44.17	**25.16**
	ARIMA	0.33	0.991	0.78	0.95	0.40	86.13	39.61	37.37
	Holt-Winters	0.34	**0.992**	**0.77**	0.95	0.42	78.02	36.55	34.05
no trend and annual seasonality (#2, #8, #14, #20, #26, #32, #38)	CUSUM	**0.82**	0.81	0.38	**0.99**	0.45	102.76	**39.17**	**28.37**
	EARS C3	0.59	0.93	0.47	0.97	**0.48**	**72.44**	55.60	30.05
	ARIMA	0.30	**0.9922**	**0.81**	0.94	0.38	95.08	48.50	45.75
	Holt-Winters	0.33	0.9921	0.79	0.95	0.41	86.78	45.87	42.65
no trend and biannual seasonality (#3, #9, #15, #21, #27, #33, #39)	CUSUM	**0.85**	0.78	0.37	**0.99**	0.45	110.00	**54.44**	**34.63**
	EARS C3	0.62	0.91	0.44	0.97	**0.46**	**80.18**	85.66	43.60
	ARIMA	0.27	**0.9940**	**0.85**	0.94	0.36	103.96	71.81	67.72
	Holt-Winters	0.31	0.9935	0.82	0.94	0.39	97.16	69.63	64.50
trend and no seasonality (#4, #10, #16, #22, #28, #34, #40)	CUSUM	**0.67**	0.84	0.34	**0.98**	0.38	82.08	**39.40**	**31.78**
	EARS C3	0.48	0.94	0.42	0.96	**0.42**	**56.70**	42.49	26.06
	ARIMA	0.31	0.97	**0.63**	0.95	0.35	66.95	43.27	40.37
	Holt-Winters	0.30	**0.98**	0.62	0.95	0.35	60.88	38.28	35.58
trend and annual seasonality (#5, #11, #17, #23, #29, #35, #41)	CUSUM	**0.73**	0.84	0.40	**0.99**	**0.44**	86.68	**44.79**	**34.32**
	EARS C3	0.55	0.91	0.38	0.97	0.42	**61.68**	50.87	29.73
	ARIMA	0.25	0.9761	**0.65**	0.94	0.30	77.71	53.68	50.28
	Holt-Winters	0.28	**0.9771**	0.63	0.94	0.33	69.82	47.60	44.17
trend and biannual seasonality (#6, #12, #18, #24, #30, #36, #42)	CUSUM	**0.77**	0.76	0.29	**0.99**	0.37	98.25	**60.10**	**41.15**
	EARS C3	0.57	0.87	0.32	0.96	**0.39**	**71.43**	75.41	42.05
	ARIMA	0.21	0.977	0.66	0.93	0.27	87.60	77.75	73.03
	Holt-Winters	0.28	**0.979**	**0.68**	0.94	0.34	80.25	69.99	65.00

**Note:** Bold-faced values indicate the best average values.

**Table 2:** The comparison of average performances for each baseline frequency and dispersion parameter.

Baseline Frequency and Dispersion’s Parameter	Method	Sensitivity	Specificity	PPV	NPV	F1 Score	sMAPE	RMSE	MAD
θ = 0.1 and ϕ = 1.5 (#1, #2, #3, #4, #5, #6)	CUSUM	**0.63**	0.89	0.39	**0.98**	0.38	99.29	7.74	6.79
	EARS C3	0.47	0.90	0.26	0.97	0.32	**73.02**	5.98	**4.75**
	ARIMA	0.34	0.978	0.54	0.96	0.38	88.35	7.10	6.60
	Holt-Winters	0.40	**0.981**	**0.60**	0.96	**0.43**	77.71	**5.62**	5.23
θ = −2 and ϕ = 2 (#7, #8, #9, #10, #11, #12)	CUSUM	0.22	0.946	0.15	**0.976**	0.13	78.59	4.24	3.94
	EARS C3	0.11	**0.951**	**0.21**	0.974	0.08	70.74	4.17	3.70
	ARIMA	**0.27**	0.942	0.18	0.976	**0.18**	55.75	2.85	2.63
	Holt-Winters	0.25	0.948	0.18	0.976	0.18	**42.17**	**2.35**	**2.15**
θ = 1.5 and ϕ = 1 (#13, #14, #15, #16, #17, #18)	CUSUM	**0.78**	0.84	0.37	**0.98**	0.44	84.37	16.92	14.88
	EARS C3	0.57	0.89	0.33	0.97	0.40	**62.95**	14.63	**11.78**
	ARIMA	0.33	0.98	0.65	0.95	0.40	76.65	16.72	15.41
	Holt-Winters	0.39	**0.98**	**0.71**	0.96	**0.46**	68.76	**13.41**	12.40
θ = 0.5 and ϕ = 5 (#19, #20, #21, #22, #23, #24)	CUSUM	**0.92**	0.78	0.32	**0.99**	0.44	128.32	**13.48**	**10.24**
	EARS C3	0.66	0.91	0.41	0.97	**0.49**	**106.98**	16.14	10.86
	ARIMA	0.34	0.99	0.73	0.95	0.43	130.47	18.60	17.61
	Holt-Winters	0.39	**0.99**	**0.81**	0.95	0.49	125.34	16.64	15.65
θ = 2.5 and ϕ = 3 (#25, #26, #27, #28, #29, #30)	CUSUM	**0.95**	0.72	0.39	**0.99**	0.51	123.72	**79.86**	**44.25**
	EARS C3	0.72	0.87	0.45	0.96	**0.53**	**87.95**	118.84	60.48
	ARIMA	0.22	**1.00**	**1.00**	0.92	0.34	129.97	119.38	112.79
	Holt-Winters	0.24	1.00	0.88	0.92	0.35	123.99	114.27	106.04
θ = 3.75 and ϕ = 1.1 (#31, #32, #33, #34, #35, #36)	CUSUM	**0.91**	0.80	0.46	**0.99**	0.54	71.96	49.27	40.82
	EARS C3	0.70	0.93	0.57	0.97	**0.60**	**38.16**	49.78	**30.90**
	ARIMA	0.26	**1.00**	**1.00**	0.93	0.39	56.38	50.48	47.58
	Holt-Winters	0.31	1.00	0.98	0.93	0.44	52.71	**47.09**	44.08
θ = 5 and ϕ = 1.2 (#37, #38, #39, #40, #41, #42)	CUSUM	**0.97**	0.70	0.42	**1.00**	0.54	84.05	**144.57**	107.16
	EARS C3	0.70	0.96	0.72	0.96	**0.69**	**37.84**	174.86	**92.34**
	ARIMA	0.17	**1.00**	**1.00**	0.91	0.29	66.10	175.26	164.30
	Holt-Winters	0.18	0.99	0.86	0.91	0.29	61.06	159.86	148.06

**Note:** Bold-faced values indicate the best average values.

**Table 3:** The results of syndromic diarrhea surveillance data for each method.

Method Name	Daily Data			Weekly Data		
	sMAPE	RMSE	MAD	sMAPE	RMSE	MAD
CUSUM (standard)	115.43	99.00	82.78	75.62	468.32	369.62
CUSUM (rossi)	102.58	94.23	78.10	61.07	417.77	332.98
CUSUM (glm with trend)	91.29	92.05	78.57	33.16	287.27	248.25
CUSUM (rossi and glm with trend)	79.23	**86.60**	**74.09**	31.27	**276.17**	**240.29**
EARS C1	59.47	104.36	91.01	34.43	429.84	330.23
EARS C2	59.71	106.52	91.89	36.23	436.10	344.79
EARS C3	**53.35**	86.84	78.57	**30.84**	337.40	268.97
ARIMA	66.07	107.56	101.38	44.95	449.46	429.74
Holt-Winters	62.72	99.65	93.57	36.63	349.30	335.33

**Note:** Bold-faced values indicate the best average values.

## Supplementary Materials

The following are available online at http://www.mdpi.com/1660-4601/15/5/966/s1. Table S1: 42 scenarios used to generate data, Table S2: The average result of the best CUSUM (glm with trend) algorithm, Table S3: The average result of the EARS C3 algorithm, Table S4: The result of the ARIMA algorithm, Table S5: The result of Holt-Winter algorithm, Figure S1: Daily syndromic diarrhea surveillance data, Figure S2: Weekly syndromic diarrhea surveillance data, Figure S3: The results of the selected algorithms for daily real data, Figure S4: The results of the selected algorithms for weekly real data.
