# Supplementary material for: A Simulation-Based Study on the Comparison of Statistical and Time Series Forecasting Methods for Early Detection of Infectious Disease Outbreaks
Source: Int J Environ Res Public Health. 2018 May 11;15(5):966. doi: 10.3390/ijerph15050966 (PMC5982005; doi:10.3390/ijerph15050966)
Supplement: Supplementary file 1 [file ijerph-15-00966-s001.pdf]

Supplemental Materials

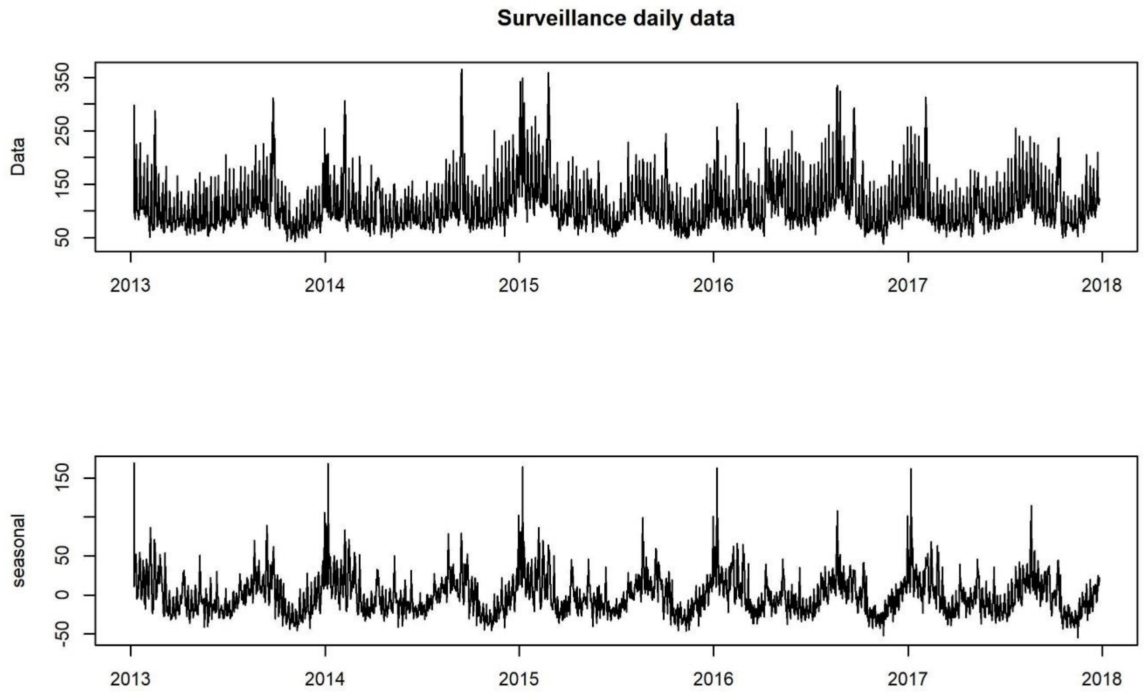

Figure S1. Daily Diarrhea syndromic surveillance data.

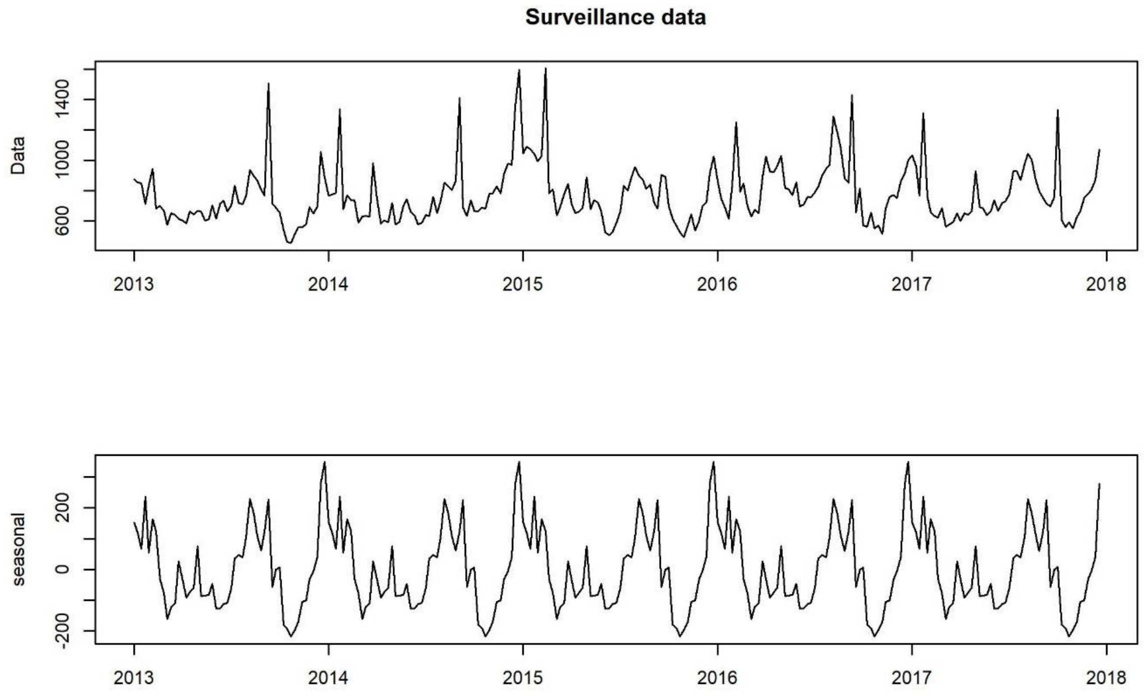

Figure S2. Weekly Diarrhea syndromic surveillance data.

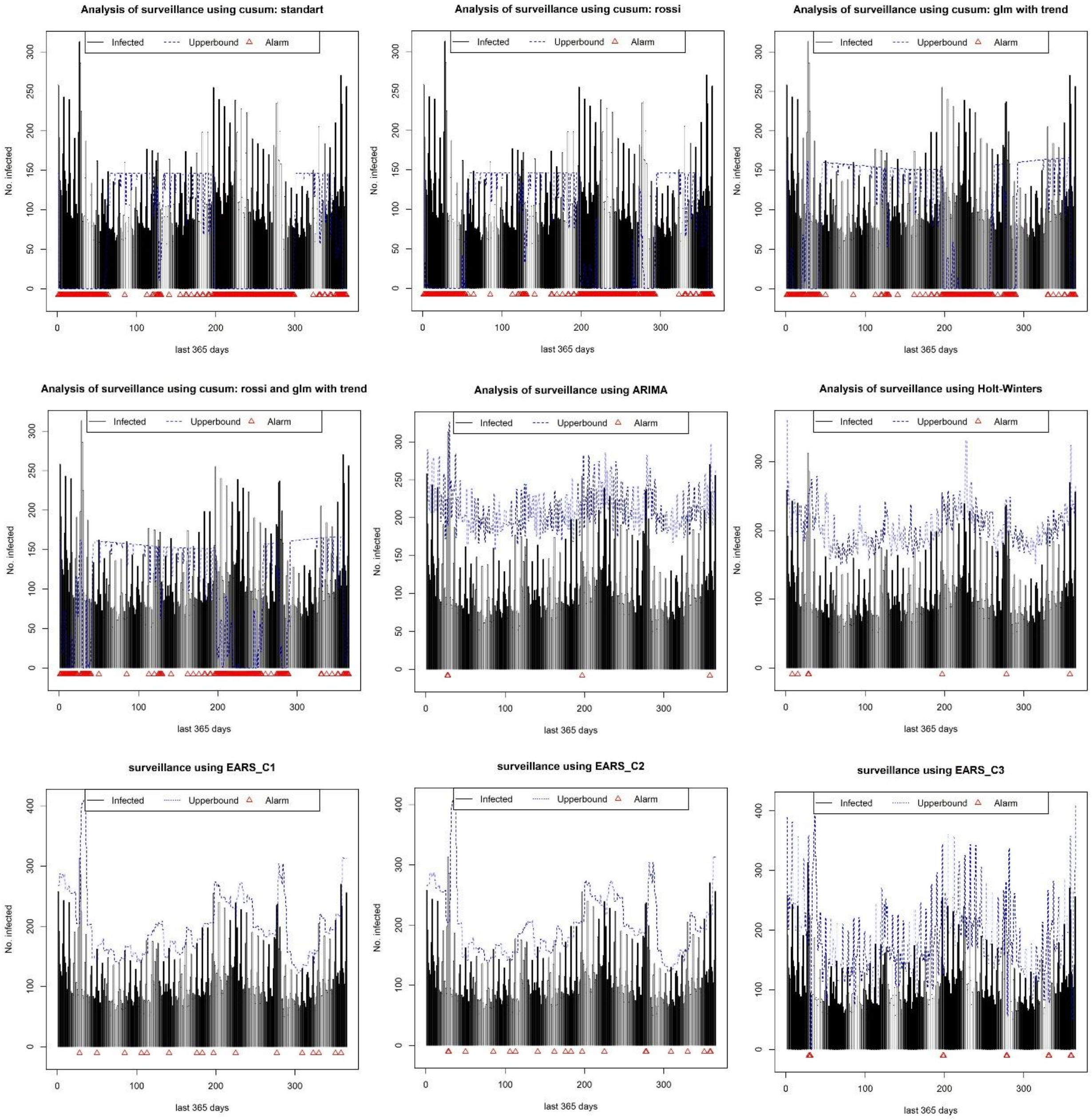

Figure S3. The results of the selected algorithms for daily real data.

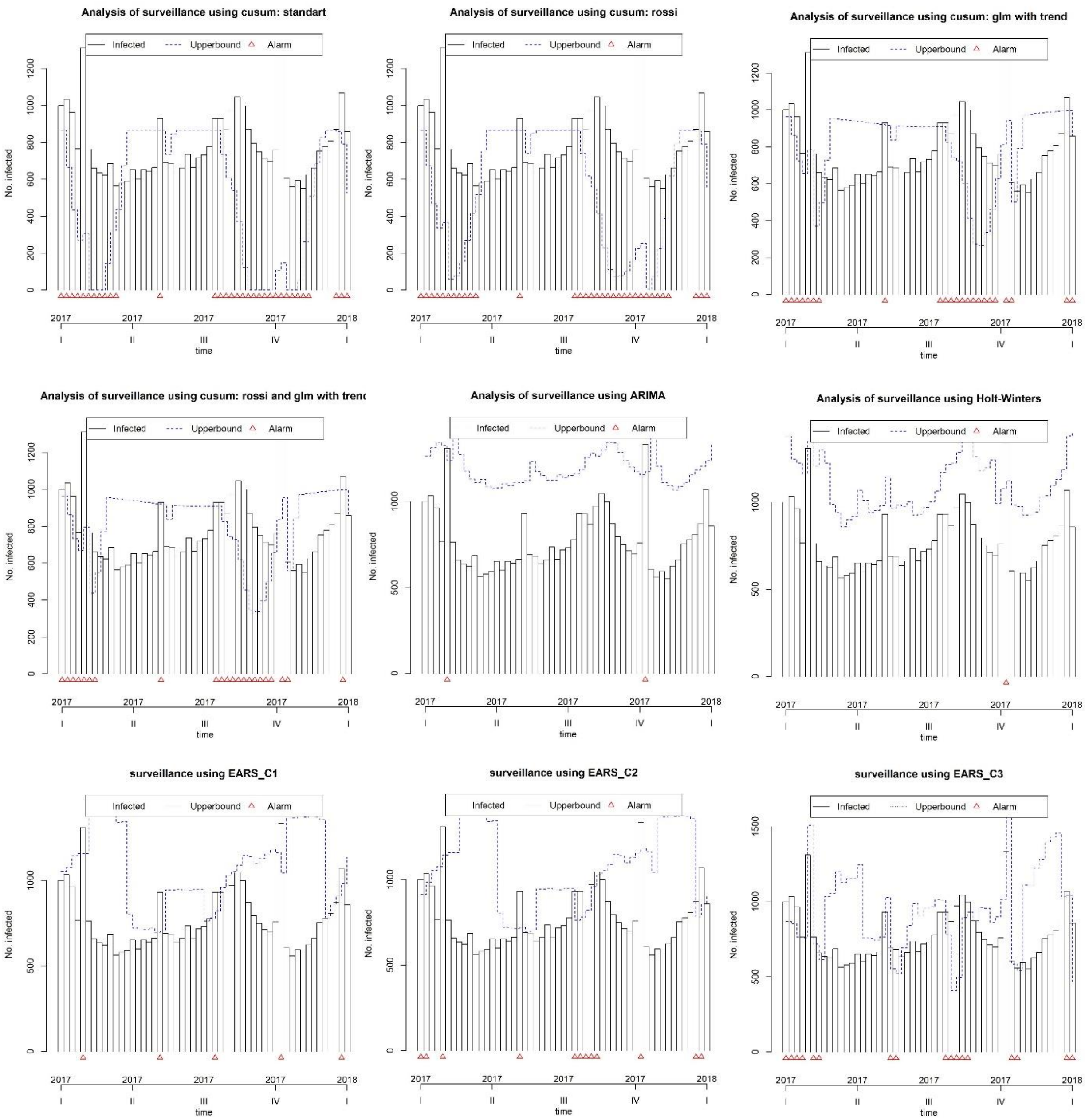

Figure S4. The results of the selected algorithms for weekly real data.

Table S1. 42 scenarios used to generate data.

Scenario	θ	β	γ_1	γ_2	ϕ	m	Trend
1	0.1	0	0	0	1.5	0	0
2	0.1	0	0.6	0.6	1.5	1	0
3	0.1	0	0.6	0.6	1.5	2	0
4	0.1	0.0025	0	0	1.5	0	1
5	0.1	0.0025	0.6	0.6	1.5	1	1
6	0.1	0.0025	0.6	0.6	1.5	2	1
7	-2	0	0	0	2	0	0
8	-2	0	0.1	0.3	2	1	0
9	-2	0	0.1	0.3	2	2	0
10	-2	0.005	0	0	2	0	1
11	-2	0.005	0.1	0.3	2	1	1
12	-2	0.005	0.1	0.3	2	2	1
13	1.5	0	0	0	1	0	0
14	1.5	0	0.2	-0.4	1	1	0
15	1.5	0	0.2	-0.4	1	2	0
16	1.5	0.003	0	0	1	0	1
17	1.5	0.003	0.2	-0.4	1	1	1
18	1.5	0.003	0.2	-0.4	1	2	1
19	0.5	0	0	0	5	0	0
20	0.5	0	0.5	0.5	5	1	0
21	0.5	0	0.5	0.5	5	2	0
22	0.5	0.002	0	0	5	0	1
23	0.5	0.002	0.5	0.5	5	1	1
24	0.5	0.002	0.5	0.5	5	2	1
25	2.5	0	0	0	3	0	0
26	2.5	0	1	0.1	3	1	0
27	2.5	0	1	0.1	3	2	0
28	2.5	0.001	0	0	3	0	1
29	2.5	0.001	1	0.1	3	1	1
30	2.5	0.001	1	0.1	3	2	1
31	3.75	0	0	0	1.1	0	0
32	3.75	0	0.1	-0.1	1.1	1	0
33	3.75	0	0.1	-0.1	1.1	2	0
34	3.75	0.001	0	0	1.1	0	1
35	3.75	0.001	0.1	-0.1	1.1	1	1
36	3.75	0.001	0.1	-0.1	1.1	2	1
37	5	0	0	0	1.2	0	0
38	5	0	0.05	0.01	1.2	1	0
39	5	0	0.05	0.01	1.2	2	0
40	5	0.0001	0	0	1.2	0	1
41	5	0.0001	0.05	0.01	1.2	1	1
42	5	0.0001	0.05	0.01	1.2	2	1

Table S2. The average result of the best CUSUM (glm with trend) algorithm.

Scenario	Sensitivity	Specificity	PPV	NPV	F measure	sMAPE	RMSE	MAD
1	0.53	0.95	0.46	0.98	0.39	98.02	4.56	4.26
2	0.77	0.91	0.43	0.99	0.48	106.82	5.26	4.66
3	0.85	0.83	0.35	0.99	0.44	120.31	6.97	5.66
4	0.28	0.97	0.37	0.97	0.23	79.30	8.62	8.05
5	0.56	0.95	0.54	0.98	0.42	84.47	9.38	8.46
6	0.79	0.74	0.22	0.98	0.32	106.82	11.67	9.66
7	0.29	0.95	0.22	0.98	0.18	64.79	1.85	1.74
8	0.35	0.95	0.23	0.98	0.21	66.32	1.90	1.79
9	0.42	0.95	0.29	0.98	0.25	68.97	1.94	1.83
10	0.08	0.95	0.06	0.97	0.04	90.00	6.60	6.12
11	0.08	0.95	0.05	0.97	0.04	89.58	6.54	6.04
12	0.13	0.93	0.07	0.97	0.06	91.88	6.63	6.10
13	0.87	0.87	0.43	0.99	0.51	103.66	10.19	9.00
14	0.86	0.87	0.41	0.99	0.50	104.63	10.79	9.27
15	0.85	0.82	0.33	0.99	0.42	108.42	11.53	9.63
16	0.64	0.91	0.41	0.97	0.43	54.26	20.54	18.62
17	0.73	0.92	0.50	0.98	0.52	55.25	21.34	19.13
18	0.74	0.67	0.15	0.97	0.24	80.00	27.15	23.65
19	0.91	0.81	0.30	0.99	0.42	131.14	7.88	6.48
20	0.93	0.79	0.32	1.00	0.45	138.22	10.59	8.00
21	0.98	0.74	0.34	1.00	0.47	150.92	17.41	11.02
22	0.90	0.80	0.30	0.99	0.43	108.35	11.60	9.96
23	0.90	0.81	0.33	0.99	0.45	116.31	14.45	11.82
24	0.91	0.75	0.31	0.99	0.42	125.01	18.96	14.18
25	0.94	0.77	0.37	0.99	0.49	108.41	26.24	19.29
26	0.98	0.72	0.42	1.00	0.54	132.05	60.20	33.28
27	0.97	0.67	0.40	0.99	0.51	151.50	145.46	68.48
28	0.90	0.78	0.36	0.99	0.46	93.51	30.57	24.38
29	0.96	0.75	0.44	0.99	0.55	114.79	62.00	39.29
30	0.97	0.66	0.36	0.99	0.49	142.07	154.67	80.75
31	0.89	0.81	0.47	0.99	0.53	77.67	41.42	34.31
32	0.91	0.75	0.42	0.99	0.50	84.93	44.59	35.81
33	0.92	0.76	0.43	0.99	0.51	85.05	45.91	36.89
34	0.95	0.83	0.50	1.00	0.60	60.07	51.95	44.52
35	0.89	0.84	0.50	0.99	0.57	60.96	54.48	45.88
36	0.89	0.82	0.47	0.99	0.54	63.04	57.28	47.49
37	0.99	0.73	0.43	1.00	0.56	79.78	139.16	101.61
38	0.97	0.68	0.40	1.00	0.51	86.32	140.84	105.76
39	0.96	0.72	0.46	0.99	0.57	84.81	151.83	108.91
40	0.97	0.65	0.38	0.99	0.50	89.09	145.94	110.80
41	0.98	0.69	0.42	1.00	0.54	85.40	145.32	109.63
42	0.97	0.73	0.43	1.00	0.55	78.91	144.36	106.23

Table S3. The average result of the EARS C3 algorithm.

Scenario	Sensitivity	Specificity	PPV	NPV	F measure	sMAPE	RMSE	MAD
1	0.4	0.94	0.29	0.97	0.33	67.78	2.79	2.36
2	0.56	0.93	0.35	0.97	0.42	77.25	4.04	3.08
3	0.64	0.89	0.32	0.97	0.41	88.08	6.84	4.49
4	0.22	0.93	0.13	0.96	0.16	56.16	5.14	4.67
5	0.5	0.9	0.24	0.97	0.31	65.24	6.42	5.5
6	0.52	0.83	0.2	0.96	0.28	83.6	10.66	8.38
7	0.15	0.99	0.45	0.98	0.14	NA	NA	NA
8	0.07	0.99	0.32	0.97	0.08	NA	NA	NA
9	0.11	1	0.34	0.98	0.11	NA	NA	NA
10	0.08	0.92	0.03	0.97	0.04	69.81	4.15	3.68
11	0.11	0.91	0.04	0.97	0.06	69.89	4.15	3.68
12	0.15	0.9	0.05	0.97	0.07	72.52	4.23	3.74
13	0.63	0.92	0.41	0.97	0.48	77.09	9.81	7.35
14	0.67	0.91	0.4	0.98	0.48	77.97	10.43	7.48
15	0.67	0.89	0.33	0.97	0.43	82.75	11.9	8.41
16	0.45	0.94	0.37	0.96	0.37	40.44	16.13	14.16
17	0.53	0.9	0.3	0.96	0.37	44.59	17.45	15.1
18	0.48	0.81	0.17	0.95	0.25	54.87	22.06	18.2
19	0.62	0.93	0.42	0.97	0.48	109.97	9.14	6.41
20	0.69	0.93	0.47	0.98	0.54	110.7	12.41	8.01
21	0.74	0.92	0.51	0.98	0.57	113.87	23.44	12.61
22	0.57	0.91	0.33	0.97	0.41	97.05	12.44	10.2
23	0.62	0.9	0.38	0.97	0.45	101.98	16.85	12.49
24	0.71	0.88	0.36	0.97	0.46	108.34	22.53	15.45
25	0.72	0.93	0.56	0.97	0.61	76.12	33.74	21.04
26	0.75	0.87	0.43	0.97	0.53	89.11	88.21	43.43
27	0.74	0.81	0.35	0.96	0.47	111.8	229.95	106.17
28	0.65	0.95	0.59	0.97	0.59	62.88	36.1	24.6
29	0.73	0.86	0.41	0.97	0.51	79.53	86.88	46.81
30	0.72	0.81	0.35	0.95	0.46	108.27	238.19	120.81
31	0.68	0.95	0.63	0.97	0.63	40.75	43.13	26.54
32	0.71	0.94	0.62	0.97	0.64	42.34	49.4	29.01
33	0.73	0.92	0.51	0.97	0.59	45.22	52.54	31.07
34	0.68	0.96	0.71	0.97	0.67	32.61	48.96	31.94
35	0.66	0.93	0.52	0.96	0.56	33.05	50.83	32.71
36	0.73	0.89	0.44	0.97	0.54	35.01	53.79	34.14
37	0.7	0.96	0.76	0.96	0.7	37.59	166.41	87.25
38	0.69	0.96	0.71	0.96	0.68	37.26	169.08	89.31
39	0.69	0.95	0.69	0.96	0.67	39.36	189.27	98.86
40	0.71	0.97	0.76	0.96	0.72	37.94	174.48	93.15
41	0.7	0.96	0.75	0.96	0.7	37.49	173.52	91.82
42	0.71	0.95	0.68	0.96	0.67	37.4	176.42	93.62

Table S4. The result of the ARIMA algorithm.

Scenario	Sensitivity	Specificity	PPV	NPV	F Measure	sMAPE	RMSE	MAD
1	0.39	0.98	0.54	0.97	0.40	86.38	3.96	3.75
2	0.42	0.99	0.69	0.96	0.48	99.28	5.23	4.98
3	0.34	0.99	0.82	0.96	0.44	117.29	7.89	7.51
4	0.31	0.96	0.29	0.97	0.27	60.86	5.91	5.37
5	0.33	0.97	0.43	0.96	0.35	75.01	8.05	7.37
6	0.27	0.97	0.47	0.95	0.31	91.27	11.56	10.65
7	0.30	0.97	0.28	0.98	0.25	42.44	1.50	1.45
8	0.34	0.97	0.27	0.98	0.26	43.23	1.52	1.47
9	0.37	0.97	0.32	0.98	0.30	44.56	1.54	1.50
10	0.23	0.92	0.08	0.97	0.11	66.86	4.08	3.70
11	0.17	0.91	0.05	0.97	0.08	67.90	4.15	3.76
12	0.19	0.91	0.08	0.97	0.10	69.53	4.28	3.88
13	0.41	1.00	0.90	0.96	0.52	99.62	11.95	11.38
14	0.36	1.00	0.89	0.95	0.47	104.77	13.38	12.71
15	0.35	0.99	0.84	0.95	0.46	106.86	14.38	13.66
16	0.38	0.97	0.51	0.95	0.40	43.04	16.99	15.38
17	0.29	0.97	0.44	0.95	0.33	50.04	20.31	18.33
18	0.22	0.96	0.34	0.94	0.25	55.58	23.32	20.99
19	0.39	0.99	0.70	0.96	0.47	133.71	11.22	10.76
20	0.35	0.99	0.83	0.95	0.46	142.84	15.48	14.83
21	0.31	1.00	0.98	0.94	0.44	156.49	25.49	24.03
22	0.42	0.97	0.53	0.96	0.44	105.12	14.20	13.29
23	0.33	0.98	0.64	0.94	0.41	115.86	18.57	17.51
24	0.27	0.99	0.73	0.94	0.37	128.78	26.66	25.26
25	0.32	1.00	1.00	0.94	0.47	111.61	37.84	36.01
26	0.19	1.00	1.00	0.92	0.31	141.95	87.49	82.37
27	0.15	1.00	1.00	0.90	0.25	164.49	221.65	209.94
28	0.33	1.00	0.98	0.94	0.47	85.66	38.00	35.97
29	0.18	1.00	1.00	0.92	0.30	123.30	92.52	87.14
30	0.16	1.00	1.00	0.90	0.27	152.84	238.75	225.32
31	0.28	1.00	1.00	0.93	0.41	64.61	46.71	44.25
32	0.25	1.00	1.00	0.93	0.38	68.10	51.05	48.06
33	0.21	1.00	1.00	0.92	0.34	69.79	52.87	49.84
34	0.32	1.00	1.00	0.93	0.47	42.48	46.92	44.34
35	0.26	1.00	0.98	0.93	0.39	45.53	51.17	48.05
36	0.20	1.00	0.99	0.93	0.32	47.77	54.15	50.95
37	0.19	1.00	1.00	0.91	0.31	64.57	164.12	153.96
38	0.18	1.00	1.00	0.91	0.30	65.41	165.35	155.85
39	0.16	1.00	1.00	0.90	0.27	68.21	178.83	167.54
40	0.17	1.00	1.00	0.90	0.28	64.63	176.77	164.52
41	0.17	1.00	1.00	0.91	0.28	66.33	180.95	169.81
42	0.17	1.00	1.00	0.91	0.28	67.41	185.55	174.12

Table S5. The result of Holt-Winter algorithm.

Scenario	Sensitivity	Specificity	PPV	NPV	F Measure	sMAPE	RMSE	MAD
1	0.35	0.99	0.55	0.97	0.37	75.09	3.29	3.10
2	0.47	0.99	0.79	0.97	0.54	87.72	4.28	4.05
3	0.46	1.00	0.91	0.96	0.57	109.35	6.84	6.48
4	0.26	0.96	0.29	0.96	0.24	53.13	4.97	4.50
5	0.39	0.97	0.45	0.96	0.39	62.57	5.95	5.45
6	0.44	0.98	0.62	0.96	0.49	78.41	8.36	7.80
7	0.27	0.97	0.31	0.98	0.23	25.02	1.24	1.19
8	0.32	0.97	0.27	0.98	0.25	26.20	1.26	1.21
9	0.35	0.97	0.32	0.98	0.28	27.00	1.28	1.23
10	0.20	0.93	0.08	0.97	0.11	57.99	3.44	3.09
11	0.14	0.92	0.05	0.97	0.07	57.78	3.40	3.06
12	0.21	0.92	0.09	0.97	0.11	59.02	3.47	3.13
13	0.44	1.00	0.95	0.96	0.56	92.22	10.31	9.79
14	0.43	1.00	0.92	0.96	0.55	97.15	11.41	10.83
15	0.41	1.00	0.94	0.96	0.53	99.44	12.28	11.66
16	0.35	0.97	0.53	0.95	0.39	37.79	14.54	13.14
17	0.35	0.97	0.51	0.95	0.38	41.20	15.42	13.96
18	0.33	0.96	0.41	0.95	0.35	44.75	16.51	15.02
19	0.43	0.99	0.76	0.96	0.51	127.28	9.67	9.22
20	0.40	1.00	0.91	0.95	0.53	138.25	13.88	13.18
21	0.34	1.00	0.96	0.94	0.47	153.77	23.85	22.27
22	0.43	0.97	0.59	0.96	0.47	97.60	12.05	11.19
23	0.39	0.99	0.72	0.95	0.48	110.19	16.18	15.23
24	0.34	1.00	0.89	0.94	0.46	124.94	24.21	22.79
25	0.37	1.00	0.99	0.94	0.51	105.84	34.23	32.26
26	0.19	0.99	0.81	0.92	0.30	132.57	85.94	79.14
27	0.16	0.99	0.83	0.90	0.25	160.36	221.76	205.68
28	0.34	1.00	0.98	0.94	0.48	81.40	35.31	33.31
29	0.18	0.99	0.84	0.92	0.29	114.75	85.67	79.39
30	0.17	0.99	0.86	0.90	0.28	149.00	222.73	206.48
31	0.31	1.00	0.98	0.94	0.43	60.08	43.06	40.33
32	0.28	1.00	0.97	0.93	0.41	63.73	47.21	44.18
33	0.26	1.00	0.95	0.93	0.39	65.45	49.69	46.18
34	0.36	1.00	0.98	0.94	0.50	40.18	44.59	41.89
35	0.31	1.00	1.00	0.93	0.45	42.50	47.73	44.77
36	0.31	1.00	0.99	0.94	0.45	44.32	50.28	47.14
37	0.19	0.99	0.87	0.91	0.30	60.63	154.03	142.45
38	0.18	0.99	0.85	0.91	0.29	61.86	157.11	145.97
39	0.17	0.99	0.85	0.90	0.28	64.76	171.70	158.03
40	0.18	1.00	0.89	0.90	0.29	58.10	153.06	141.91
41	0.18	0.99	0.85	0.91	0.29	59.73	158.83	147.32
42	0.17	1.00	0.86	0.91	0.27	61.31	164.40	152.66
